# Connection to mental health care upon community reentry for detained youth: a qualitative study

**DOI:** 10.1186/1471-2458-14-117

**Published:** 2014-02-05

**Authors:** Matthew C Aalsma, James R Brown, Evan D Holloway, Mary A Ott

**Affiliations:** 1 Department of Pediatrics, Section of Adolescent Medicine, Indiana University School of Medicine, Indianapolis, IN, USA; 2 Department of Social Work, University of Wisconsin-Oshkosh, Oshkosh, WI, USA; 3 Department of Psychology, Fordham University, Bronx, NY, USA

**Keywords:** Juvenile justice, Detention, Mental health screening, Mental health care utilization, Qualitative research

## Abstract

**Background:**

Although detained youth evidence increased rates of mental illness, relatively few adolescents utilize mental health care upon release from detention. Thus, the goal of this study is to understand the process of mental health care engagement upon community reentry for mentally-ill detained youth.

**Methods:**

Qualitative interviews were conducted with 19 youth and caregiver dyads (39 participants) recruited from four Midwest counties affiliated with a state-wide mental health screening project. Previously detained youth (ages 11–17), who had elevated scores on a validated mental health screening measure, and a caregiver were interviewed 30 days post release. A critical realist perspective was used to identify themes on the detention and reentry experiences that impacted youth mental health care acquisition.

**Results:**

Youth perceived detention as a crisis event and having detention-based mental health care increased their motivation to seek mental health care at reentry. Caregivers described receiving very little information regarding their child during detention and felt “out of the loop,” which resulted in mental health care utilization difficulty. Upon community reentry, long wait periods between detention release and initial contact with court or probation officers were associated with decreased motivation for youth to seek care. However, systemic coordination between the family, court and mental health system facilitated mental health care connection.

**Conclusions:**

Utilizing mental health care services can be a daunting process, particularly for youth upon community reentry from detention. The current study illustrates that individual, family-specific and systemic issues interact to facilitate or impair mental health care utilization. As such, in order to aid youth in accessing mental health care at detention release, systemic coordination efforts are necessary. The systematic coordination among caregivers, youth, and individuals within the justice system are needed to reduce barriers given that utilization of mental health care is a complex process.

## Background

Each year, approximately 2 million U.S. youth are arrested and held in a detention or correctional facility [1]. Virtually all detained youth will return to their community and face the need to reintegrate with families, communities, and society [2]. Detained youth have high rates of psychopathology [3,4] and evidence suggests that connection to effective mental health treatment results in decreased recidivism rates [5-8]. Central to engagement in care is mental health screening of detained youth [9]. Benefits of mental health screening include better identification of youth with mental health needs, improving care during detention, and increased communication between staff and youth [10]. However, mere identification of mental health needs does not necessarily lead to engagement in mental health care, or to successful community re-integration [11,12]. There continues to be a significant disconnect between the identification of detained youth with psychopathology and connection to appropriate community mental health care. Although few studies of mental health usage rates exist, one study found that only 8% of youth leaving a detention facility with a documented need for mental health treatment received services in the community within six months of release [13].

### Barriers to mental health care utilization

Little data exist on facilitators and barriers to mental health care utilization among detained youth and their caregivers. One study identified individual barriers for youth such as being unsure of where to go for care, mental health stigma, and concern about cost of care. Additionally, when youth did receive mental health care services in the past, they felt the services were ineffective and unhelpful in achieving life goals [14]. Systemic barriers have also been identified, such as probation officers’ knowledge of mental health and the availability of mental health professionals [15]. Although these studies provide an important foundation, they have focused on youth or professional perspectives, rather than youth-caregiver dyads.

Recently detained adolescents with mental health problems are a vulnerable, and often hidden, population, particularly at the time of community re-entry. Little is known, however, about the experiences of detained youth, much less their caregivers, during community reentry. Therefore, the goal of this study was to explore the perceptions of youth and their caregivers in accessing mental health care at the time of community reentry. Given the paucity of research in this area, we chose a qualitative research method.

## Methods

### Participants and recruitment

This study was reviewed, and approved, by the full Indiana University Institutional Review Board. To maximize recruitment while maintaining protections for this vulnerable population, we used a multi-step approach. We identified 11–17 year olds from four counties’ court records who had 1) scored above the cutoff on the Massachusetts Youth Screening Instrument-2 (MAYSI-2), and 2) had been released 30 or more days earlier. Participating counties were selected due to geographical and demographical diversity in an effort to reduce sampling bias. Further, each county was participating in a state-wide mental health screening initiative, which employed the MAYSI-2 at intake to detention. Thirty days was chosen as a meaningful time frame for youth to connect to mental health care upon community reentry.

The MAYSI-2 is a 52-item, dichotomous validated mental health screening instrument used to identify youth who warrant further mental health assessment [3,16,17]. A positive screen on the MAYSI-2 consists of a score of a caution or warning range (2 or higher) on the 5-item suicide ideation scale, or a warning range score (3 or higher) on two or more of the remaining six subscales (alcohol/drug use, angry/irritable, depressed/anxious, somatic complaints, thought disturbance, traumatic experiences) [3].

Detention center staff called caregivers of youth who met inclusion criteria and read a script describing the study, after which caregivers were asked if they would be interested in receiving additional study information. Interested caregivers were contacted by research staff, who explained the study and reviewed the inclusion criteria: (a) ages 11 and 17 years; (b) a positive screen on their initial MAYSI-2; (c) released back to their home; (d) released for a minimum of 30 days; and (e) having at least one caregiver (parent, grandparent or legal guardian) willing to be interviewed. Interested caregivers and youth scheduled an interview at a location of the caregiver’s choosing, usually their home, but the option was offered to meet in a private room at a public library or the detention facility itself. At the scheduled appointment, the study was re-explained, consent was obtained from both the caregiver and youth, and the interview was completed. Both caregiver and adolescent received a $20 gift card.

### Interviews

The semi-structured interviews were conducted from December 2009 to April 2010 by three doctoral-level researchers experienced in qualitative interviewing. Youth and their caregiver were interviewed separately. Youth interviews ranged from 6 to 39 minutes (M = 21.74, SD = 8.54) and caregiver interviews ranged from 17 to 51 minutes (M = 31.68, SD = 10.19). Interview guides gathered information on facilitators and barriers to engagement in mental health care. Questions focused on the youth’s and caregiver’s experiences while the youth was in detention, the process of community reentry, court sentencing, and connection to mental health services. Example questions included: “Can you describe any counseling services you received *before* you were placed in detention?”; “While in detention, were there opportunities to have counseling services?”; “What kind of support have you had in receiving counseling services *after* detention?” and “How did your probation officer affect your experience with counseling services?” Participants were asked follow-up questions to clarify and explain their responses. Each interview was digitally recorded, transcribed verbatim, and observation notes were written during and after the interviews.

### Data analysis

We used a thematic analytic approach informed by a critical realist perspective [18]. We examined the ways in which caregivers and adolescents made meaning of their detention and connection to mental health care experiences, and then how broader social contexts influenced those meanings [19]. Our goal was to understand the internal and external processes youth and caregivers experienced in connecting to mental health care. The analysis team met weekly to review interview transcripts, using an open-coding process to identify common experiences and then themes within those common experiences. Youth and caregivers had unique experiences during the detention process. Youth identified a detention experience as a crisis event and reported variable experiences with detention-based mental health care. Contemporaneously, caregivers reported feeling “out of the loop” during their child’s detention. Common experiences reported by youth and caregivers during community reentry included the critical importance of systemic coordination. We examined inter-relationships among these themes. As new interviews were completed, these themes and inter-relationships were continuously refined. Nvivo 8 software was used for data management and analysis.

## Results

### Participants

Sixty-seven caregivers had met inclusion criteria were contacted by detention staff and 43 caregivers agreed to allow research staff to describe the study to them. Twenty-seven caregivers were interested in participating in the study and agreed to be interviewed. There were eight no-shows and cancelations, resulting in a total of 19 youth-caregiver dyads or 39 participants total. For one youth, two caregivers were interviewed (see Table 1). Twelve youth were prescribed psychiatric medications, and 12 had newly received or continued with mental health services upon community reentry at the time of the interview.

**Table 1:** Demographic and contextual information

	**Youth (n = 19)**	**Caregiver (n = 20)**
Gender		
Male	12	3
Female	7	17
Age (mean)	15.5 (males)	N/A
	16 (females)	
Race/Ethnicity		
White	8	10
African American	10	8
Latino	1	0
Native American	0	1
Grade	6th – 11th	N/A
Insurance		
Medicaid	11	N/A
Private	5	
Self-pay	3	
Relationship to Youth		
Mother	N/A	14
Father		2
Mother/Father pair		1 pair (2 people)
Grandmother		2
Previous Outpatient	12	N/A
Mental Health Services		
Prescribed Psychiatric Medication	12	N/A

### Case study

A case study is offered below to describe the experiences of youth and caregivers in utilizing mental health care upon detention release. A 17-year-old Black male who had never been arrested before was detained for punching someone after getting involved in a domestic violence incident at a neighbor’s house. This youth had recently experienced a number of major life changes including a move from the South side of Chicago to a rural town in southern Indiana and losing his grandparents, who had cared for him for a number of years. Mother reported that her son’s arrest, in addition to these circumstances, prompted her to seek treatment for all of her children. This youth had never received mental health services before, although he was insured through public insurance. He reported seeking help from his high school counselor for self-identified anger problems, however, when asked if he knew where to go for mental health services he replied: “No, I would love to know where to get counseling, but I seriously don’t know.” His mother identified a counselor on her own but had been unable to get an appointment due to a lack of availability and convenient times. Regarding interaction with the court, she requested information about her son’s probation status and was told to wait for a call from the court. If she never received one, then she should assume that her son was not on probation; at the time of the interview, neither mother nor son had yet received contact from the court system. The only communication from the court was a letter informing her of a court date two months after the date of detention.

This case illustrates several common themes that were expressed by youth and their families. Below we describe each of these themes in more depth. Additionally, Table 2 includes quotes that further describe each of the below themes.

**Table 2:** Youth and caregivers’ experiences of representative quotes

**Key concept**	**Representative quote**
Caregivers felt “out of the loop” during detention	“I was not aware of any type of screening that took place and no real information once they [youth] came here. I did get a call that pretty much stated your son is here, he’s been admitted, you can talk to him for a few moments, you will be receiving information in the mail as to your court date and we’ll call you when you can come pick him up here, estimated time frame. I have no idea about any type of process that took place there.”
	-Caregiver of 15 year old male
Detention as crisis event	Interviewer: “How did your experience in juvenile detention affect you receiving counseling services?”
	Youth: “It helped me because some people, when they say they go in there, oh I just went in there because my parents told me so. Well I actually went in there with something to actually get off my chest. When I went in there it was like I had to struggle and when I had to struggle, if I tried to talk to somebody and then when I really look at it, it would be like, man they don’t really care. Then when I went to counseling she cared and then after they saw me getting better then that's when I thought that they started to care and I think me going to counseling was the biggest change that I had in my whole entire life because usually I don’t talk to nobody.”
	-13 year old female
Waiting period after re-entry- Delays in care can lead to adoption of past behaviors	“I was, like, I want to get help all I can and so I was thinking it at the time [detention], but when I got out and I started doing more stuff, I mean, like, I didn’t want to go to counselling, wake up all the time and go to counselling.”
	-14 year old male
Systemic coordination	“I do it [counseling] because I have to. My mom thinks I need it. Court thinks I need it. Everybody else thinks I need it, so why not take it then if everybody thinks it going to help?”
	-16 year old male
Systemic coordination – Key role of probation officer	Interviewer: “Did you know how to get counseling?”
	Youth: “No, my probation officer had set it up and he gave the lady my mom’s number and she called, set up and appointment, and she came and we met for the first time. She is really nice.”
	-16 year old female
Systemic coordination – Caregivers’ support	“Well originally it [my father’s attitude toward mental health care] was like a negative. I don’t care, you’re basically forcing me to go there. So I’m not even going to listen to what the dude’s saying but then me and that dude had the talk. I then talked to my dad about it and now he usually tries to be more positive when talking about it. He’ll usually let me know 20 minutes before I have to go and if he has time he’ll usually drive me. He always picks me up now but I guess after I talked to him he was basically, okay I’ll see this as a good thing instead of just you’re just a bad kid going to your counseling class because that’s what it was at first.”
	-16 year old male

### Detention – caregivers out of the loop

In general, both youth and caregivers had a difficult time describing the mental health services offered or received during detention. A common experience of caregivers was feeling “*out of the loop*” while their child was detained. For instance, caregivers did not know about the mental health screening process, results of the screening, or services their child received while in detention. The majority of caregivers desired additional information about their child during detention. This caregiver of a 13-year-old male stated:

“But I think that when they bring him to detention, I think when they’re going to assess the children; I think they should have a parent involved in there…because you just feel out of the loop at that point as a parent… They’re doing this with your child, and get a phone call a couple of days later – and oh, by the way – you know I would’ve liked to have known why they decided to put my son on a suicide watch. I mean, to me, that’s very important.”

Another caregiver of a 17-year-old male described feeling uninformed during her son’s detention stay:

Interviewer: So you didn’t hear anything about what happened when he was in detention or recommendations for you?

Caregiver: No.

Interviewer: So you’re going to go to court and just find out what happens?

Caregiver: Yeah. I asked them, I said: “Is he on probation, is there a probation officer?” You know I was asking them all these things and he just said that it would be a hearing and pretty much to show up. That was pretty much it and I was like, well what if no probation, no probation officer, no probation court. They said that someone would be calling me, if I didn’t receive a call then pretty much no.

Interviewer: So you were surprised by that?

Caregiver: Yeah.

Interviewer: What did you kind of expect?

Caregiver: Well I really didn’t know what to expect because I haven’t had -- had it done before but I do know people that have been in the system… I knew he was going to have to go court because he hasn’t been charged innocent or guilty, he’s just been charged. You still have to get his side of the story and vice versa. But I thought that -- I didn’t know it was going to be like a month or two later, I mean to me that was just pretty a long time because who’s going to remember what happened next month.

This caregiver described not being informed regarding her son’s court date or probation status, despite wanting to know what her son’s status with the court.

### Detention as crisis

Youth shared windows of opportunity to receiving mental health, sometimes just by their perception of *detention as crisis*. Describing a detention stay as a crisis event was more common for first-time offenders and youth identified with a mental health issue for the first time. Here is an example from a 17-year-old male:

Youth: When I was locked up it kind of scared me and as soon as I got there it was like, “Wow, I can’t believe I did all this and stuff. Maybe I do need counseling for all the anger problems I do have.”

Youth ascribed self-reflection, a willingness to engage with counselors, and a desire to talk to someone as a result of the crisis of detention. Several youth described increased motivation to seek out mental health care, particularly when a link came very soon after detention. Here is a 16-year-old female’s perception of receiving mental health upon community reentry:

Interviewer: What were your thoughts about having counseling after leaving detention?

Youth: Keeping it because I knew that I had hit pretty much a dead end. That I needed help to get back to where I was. The day I got out the next day, the 22nd was when I had the counseling meeting. I was thankful for that.

When engagement in mental health care did not occur soon after community reentry, youth described going back to their old patterns of behavior, such as skipping school, hanging out with negative peers, and re-engaging in the same problem behavior. This illustrates that despite a window of opportunity presenting itself to access mental health care, that window may be short lived.

### Detention-based mental health care

A number of youth reported accessing mental health services during their detention stay as very helpful. This 14-year-old male describes the available services:

Youth: Every Wednesday and Tuesday, the social worker come and she’ll interview people and then she’ll talk to you about how—are you stressed? What has been on your mind? And then you can bring up any situation you want to talk about and back in the detention, that you’ll sit in there, you’ll go in this little room, it have glass where you can look out and this door that locks… you will sit there and talk and you’ve got a certain amount of time, an hour and then the next person’s got to go.

Interviewer: Can you tell me how that—was that a helpful thing, not a helpful thing? What was your thinking about it?

Youth: It was helpful because it kept me out of my room and it was helping me get things off my chest.

This youth was able to “get things off his chest” as a result of meeting with a social worker in detention. Another 13-year-old male describes how seeing a social worker helped him get connected to services:

Interviewer: While in detention, were there ever opportunities or offers to have counseling services? While you were in detention did you see a social worker, see a counselor?

Youth: Yeah. That’s how I got into anger management [class] and stuff.

In contrast to the previous example where mental health services in detention improved day-to-day functioning during detention, for this youth, mental health services in detention led to anger management classes upon release.

### Reentry

Upon community reentry, some caregivers expressed frustration regarding the wait period after detention release and before contact with probation or receipt of a court date. For example, this caregiver of a 17-year-old male details her attempts to contact her son’s probation officer.

“I’m not picking on his probation officer, but I have called her several times in hope to find somebody for therapy for him, get his anger assessment, like this GED class they want him in, and I get no phone calls back. So no, they’ve really been no help. But if he messes up, they’re the first ones coming knocking on our door.”

### Systemic coordination

Caregivers and youth described *systemic coordination of services* as being particularly helpful in connection to mental health care. Poor coordination occurred when a caregiver was left to secure mental health care for their child without resources or information. Increased systemic coordination was evidenced when probation officers, courts, mental health providers, and family members communicated the importance of mental health counseling and provided information, referrals, and assistance in setting up mental health care. Youth and caregivers who described agreement, coordination, and assistance among key players were more likely to engage with care.

Collaboration between a caregiver and probation officer was particularly meaningful. Here is a caregiver of a 16-year-old female’s perception about coordination:

Interviewer: How did your probation officer feel about counseling?

Caregiver: How did she feel about counseling? She thought it would be good for [her].

Interviewer: She was able to communicate that clearly?

Caregiver: Uh-huh. She’s helping me with getting her back into [mental health].

Probation officers were identified as important resources for referrals and information, with great ability to facilitate systemic coordination. Most caregivers and youth saw their probation officer as having favorable attitudes toward mental health care. Here is an example of a probation officer guiding a caregiver to explore his 16-year-old youth’s need for mental health treatment:

“He actually asked us what we thought about it at one point ….basically, it was “Do you think that counseling would help him,” or was there some form of counseling you might think would work for – it was more of a question towards us I think… I think he was just kind of searching for answers, or trying to help me search for answers more than anything.”

### Model

**Figure 1:**
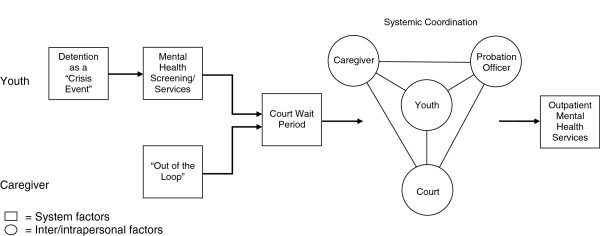
Juvenile Mental Health Care Reentry Model.

In summary, Figure 1 is a pictorial description of the overall process of detention and reentry experiences that impacted mental health care acquisition. In general, youth perceived detention as a *crisis event and* caregivers described feeling *out of the loop* during their youth’s detention. When *detention-based mental health care* was received, motivation to seek mental health care upon community reentry was increased. Upon community reentry, long wait periods between detention release and initial contact with court or probation officers were associated with decreased motivation to seek care. *Systemic coordination* between the family, court, and mental health system increased mental health care connection.

## Discussion

Utilizing mental services can be a difficult process for anyone, much less for youth recently released from juvenile detention centers. Individual, family, and system-wide factors were implicated in mental health care utilization during the reentry process. Youth described the detention stay as a crisis event. Crises that are new and emotionally salient are often a time of self-evaluation which can lead to behavior change [20]. Thus, a detention stay may represent a timely opportunity to engage otherwise resistant youth and families in mental health services. Mental health services received during detention were particularly meaningful and increased their internal motivation to seek future mental health services. This is particularly important since detained youth have reported negative experiences with previous mental health services [14].

During their youth’s detention stay, caregivers’ reported feeling “out of the loop.” Most caregivers were unaware of the mental health services their child received during detention. Given that the majority of detained youth will return to the community under caregivers’ supervision, efforts to engage caregivers during the detention stay could prove fruitful since family members have been identified as important sources of support for positive reentry of incarcerated youth [21]. Moreover, previous research has found that a “family check-up” (a motivational interviewing based approach to increase parents’ motivation and ability to recognize and intervene in their child’s risk behavior) [22] may be effective for detained and incarcerated youth [23]. At a minimum, juvenile justice personnel could communicate concerns regarding the youth’s mental health prior to release from detention and provide community resources for mental health care. Although the majority of detention centers have mental health professionals onsite [24], few provide mental services on a daily or as needed basis [25]. Moreover, some detainees are detained for short time periods, which makes communication difficult. Nonetheless, caregivers in the current study did express a desire to know about their child's mental health needs and they were also motivated to help their child receive services at community reentry.

The time period immediately after reentry was seen as important for youth and caregivers. Previous research has found the median time period between arrest and final case disposition ranged from 34–59 days. However, between 19-35% of delinquent cases exceeded 90 days with larger jurisdictions evidencing even greater waiting periods [26]. In the current study, youth and caregivers identified the time between community reentry and receiving communication from court officials as a particularly vulnerable time where families were stuck in limbo. Immediately upon reentry, many youth were motivated to change but within days, youth described returning to old habits, routines, and behaviors. Such latency wastes any motivation for behavior change created by the crisis of detention. A more expeditious court process to facilitate connection to mental health care may harness increased motivation during this critical time period. It is recommended that the juvenile justice system consider policies which limit the time between detention release and court date, while prioritizing engagement in mental health services immediately upon release.

An important system-level finding was the critical importance of coordination between the family, mental health, and juvenile justice systems. Participants described the importance of encouragement, support, and assistance with access to care from caregivers, probation officers, and court officials. Coordination among these adults is an integral part of wraparound services, with evidence suggesting a united front of multiple, supportive adults can reduce at-risk and delinquent behavior [27]. Similarly, youth reported in open this to receiving mental health services when caregivers, probation and the court had consistent messages regarding the importance of receiving services.

### Limitations

Many caregiver youth dyads that met the study inclusion criteria were not interested in participating in the research project. Thus, it is possible that the experiences of families in the current study are not representative of most juvenile justice involved youth. The current study does, however, represent the experiences of a subsample of youth with mental health difficulties and the results of this study can inform future projects.

## Conclusions

Screening for mental health problems among detained youth is an important public health initiative. Mental health screening enables appropriate identification of detained youth with significant mental health concerns. However, mental health screening alone does not lead to connection to mental health care [28]. The results of the current study highlight the fact that connection to mental health care upon community reentry can be complex, necessitating individual, family, and system-wide coordination.

There are concerns that the juvenile justice system could be, or already is, the de facto mental health system for adolescents since community-based mental health care is relatively difficult to access [29]. Thus, there are public policy concerns about providing mental health services within the juvenile justice system [30]. However, youth in the current study describe being in “crisis” during detention and were motivated to receive mental health care upon reentry. Thus, the detention stay may be a particularly salient time to provide services to increase motivation to seek care upon community reentry as well as reduce internal psychological barriers to care. Motivational interviewing (MI) is a technique designed to do just this by improving the overall motivation to receive treatment and decreasing client ambivalence towards therapy [31]. Patients receiving a motivational assessment and interview had greater participation in treatment upon entrance to a residential alcoholism treatment program compared to those patients receiving no MI [32]. Thus, MI at detention release to increase mental health care utilization may be particularly well-suited for detained youth.

Although participating counties were involved in a statewide screening program that included protocols for screening and provision of mental health services during detention, protocols for connection to care were not included. Hence, the development of system-specific protocols is encouraged in order to remedy system-level barriers. For instance, a protocol for informing caregivers of screening results, a list of community-wide resources for mental health care, and interventions focused on increasing collaboration and communication between systems could facilitate connection to mental health care. In sum, simple protocols and interventions focused on systemic coordination of care are recommended.

## Competing interests

The authors declare that they have no financial or non-financial competing interests.

## Authors’ contributions

MCA developed the study design, managed the overall project and wrote the majority of the manuscript. JRB conducted the majority of interviews and guided the qualitative analysis. EDH aided in the qualitative analysis and writing of the manuscript. MAO oversaw the qualitative data analysis and wrote the research method section. All authors read and approved the final manuscript.

## Authors’ information

MCA is a child psychologist and his research agenda is focused on the intersection of juvenile justice and public health. JRB is a social worker with an interested in vulnerable populations as well as qualitative research methodologies. EDH is a PhD student in the Forensic Clinical Psychology at Fordham University and is interested in an academic career focused on juvenile justice mental health. MAO conducts research with vulnerable adolescent populations and enjoys conducting mixed method research projects.

## Pre-publication history

The pre-publication history for this paper can be accessed here:

http://www.biomedcentral.com/1471-2458/14/117/prepub
